# Genetic Influences on Cystic Fibrosis Lung Disease Severity

**DOI:** 10.3389/fphar.2013.00040

**Published:** 2013-04-23

**Authors:** Colleen A. Weiler, Mitchell L. Drumm

**Affiliations:** ^1^Department of Pediatrics, Case Western Reserve UniversityCleveland, OH, USA; ^2^Department of Genetics and Genome Sciences, Case Western Reserve UniversityCleveland, OH, USA

**Keywords:** polymorphism, genotype, phenotype

## Abstract

Understanding the causes of variation in clinical manifestations of disease should allow for design of new or improved therapeutic strategies to treat the disease. If variation is caused by genetic differences between individuals, identifying the genes involved should present therapeutic targets, either in the proteins encoded by those genes or the pathways in which they function. The technology to identify and genotype the millions of variants present in the human genome has evolved rapidly over the past two decades. Originally only a small number of polymorphisms in a small number of subjects could be studied realistically, but speed and scope have increased nearly as dramatically as cost has decreased, making it feasible to determine genotypes of hundreds of thousands of polymorphisms in thousands of subjects. The use of such genetic technology has been applied to cystic fibrosis (CF) to identify genetic variation that alters the outcome of this single gene disorder. Candidate gene strategies to identify these variants, referred to as “modifier genes,” has yielded several genes that act in pathways known to be important in CF and for these the clinical implications are relatively clear. More recently, whole-genome surveys that probe hundreds of thousands of variants have been carried out and have identified genes and chromosomal regions for which a role in CF is not at all clear. Identification of these genes is exciting, as it provides the possibility for new areas of therapeutic development.

## Cystic Fibrosis Background

Cystic fibrosis (CF) is the most common lethal autosomal recessive disease in Caucasians, affecting an estimated 1 in 3,300 live-born infants (Davis et al., 1996). Affected individuals have variants in both copies of the 230-kb CF transmembrane conductance regulator gene (CFTR), that result in significant reduction or absence of CFTR function. The *CFTR* gene is located on the long arm of chromosome 7 at position 7q31and encodes a 1,480 amino acid protein (Riordan et al., 1989; Rommens et al., 1989) with cAMP-dependent anion channel activity (Bear et al., 1992) found in the apical membranes of epithelial cells in the lungs, olfactory sinuses, pancreas, intestines, vas deferens, and sweat ducts, as well as non-epithelial cells such as immune cells (myeloid and lymphocytes) and various muscle cell types (Yoshimura et al., 1991; Krauss et al., 1992; McDonald et al., 1992; Dong et al., 1995; Moss et al., 2000; Robert et al., 2005; Di et al., 2006; Vandebrouck et al., 2006; Divangahi et al., 2009; Lamhonwah et al., 2010). Low or absent CFTR function in the airway epithelium not only results in decreased chloride permeability, but also in increased sodium absorption across the epithelium, impairing hydration of the airway mucosal surface and resulting in thick, sticky mucus and an environment for bacteria to thrive. Thus, typical clinical features of CF include chronic infection and inflammation of the airways. Accordingly, a hallmark characteristic of the CF airways is progressive bronchiectasis; this destruction and dilation of the airways is the primary cause of morbidity and mortality of CF patients. In addition to the airway manifestations, most CF patients will experience exocrine pancreatic insufficiency, males are most often sterile, and other co-morbidities such as liver disease and diabetes are common as well. Previously considered almost exclusively a pediatric disease, CF babies now have a predicted median survival of nearly 40 years (Cystic Fibrosis Foundation Patient Registry, 2009).

## Heterogeneity of *CFTR*

To date, over 1,800 CF-associated mutations have been described^1^ and the effects of these mutations have been grouped into six general classes based on the consequence to CFTR message and/or protein (Zielenski, 2000). These range from complete absence of full-length, functional CFTR protein (class I), proteins that do not traffic to the membrane well due to misfolding (class II), proteins that reach the membrane but do not respond to activation stimuli such as phosphorylation (class III), proteins that reach the membrane and activate, but do not conduct anions sufficiently to prevent disease (class IV), mutations that reduce the amount of functional CFTR, such as by gene expression regulation or protein trafficking (class V), and proteins that are unstable and experience increased turnover in the plasma membrane (class VI). It should be noted that these classes are not mutually exclusive, as a single change may have multiple effects on the protein.

Given the diversity of mutations, it is perhaps not surprising that there is a wide range of phenotypic variability in CF simply due to variation in *CFTR*. Many reports of correlations between *CFTR* genotype and clinical phenotype exist (Kerem et al., 1990a; Stuhrmann et al., 1991; The Cystic Fibrosis Genotype-Phenotype Consortium, 1993; Tsui and Durie, 1997; Zielenski, 2000), with the most extensive catalog to date carried out as an international effort^2^ and currently includes data on over 35,000 patients. Because most CF mutations are rare, surveying such a large number of individuals makes it possible to most reliably assess the phenotypic effects associated with a genotype, rather than extrapolate from individual cases.

In addition to *CFTR* genotype, there is evidence that gender contributes to phenotypic variability (Davis, 1999). Females are reported to have a reduced median survival age (by approximately 3 years), an earlier average age of *Pseudomonas aeruginosa* infection in the lungs, greater rates of pulmonary decline, and elevated resting energy expenditure when compared to males (Demko et al., 1995; Corey et al., 1997; Allen et al., 2003). Although some current studies replicate these findings (Barr et al., 2011; Reid et al., 2011), others show no evidence of a gender gap and propose that phenotypic variability could be attributed to non-uniformity of care or the need to account for other factors such as body habitus, presence of diabetes, or the finding that females are more likely to be diagnosed later in life than males (Widerman et al., 2000; Milla et al., 2005; Rodman et al., 2005; Verma et al., 2005; Stern et al., 2008; Fogarty et al., 2012).

## Genomic Heterogeneity and Clinical Variation

Even among patients with the same *CFTR* genotype, there is a wide range of phenotypic variability (Kerem et al., 1990a; Tsui and Durie, 1997). Perhaps most notably, there is remarkable variation of pulmonary phenotype, with some patients maintaining normal lung function well into adolescence and adulthood while others do quite poorly even at a very young age (Kerem et al., 1990a). Understanding the causes of this variation is important, as it provides insight into developing new therapies, or improving existing ones.

Clearly environmental factors contribute to clinical variation; exposure to tobacco smoke, bacterial infections, and socioeconomic status have all been implicated as having detrimental effects on pulmonary phenotype of CF patients (Kerem et al., 1990b; Rubin, 1990; Corey and Farewell, 1996; Schechter et al., 2001; O’Connor et al., 2003) while improvement of nutritional status, through aggressive treatment, has been associated with improvements in pulmonary phenotype (Steinkamp and von der Hardt, 1994). Each of the environmental sources of clinical variation provide potential intervention points, but it is also clear that there are heritable sources (Mekus et al., 2000; Vanscoy et al., 2007) of variation as well and that may provide insight into even more therapeutic targets.

## Evidence of Genetic Modifiers of Disease

Human twin and sibling studies have been useful in verifying the role of modifier genes, and quantifying their contribution to phenotypic variation. Mekus et al. (2000) found in a survey of 277 sibling pairs, with 29 monozygous and 12 dizygous pairs, that a combined index of lung function and body mass was more concordant among monozygous twins (sharing 100% of genetic material) than dizygous twins or other sibling pairs (sharing 50% of genetic material), pointing to a genetic etiology of variation. Similarly, Vanscoy et al. (2007) examined the pulmonary phenotype of 57 twin pairs and 231 sibling pairs with CF. Lung function measurements were significantly more concordant between monozygous twins than dizygous twins, also indicating the presence of genetic modifiers. The similarity in lung function between sibling pairs was compared to the similarity in lung function in unrelated patients, and again was found to be more similar. Heritability estimates were calculated from these data, and it was determined that non-*CFTR* genetic variation could account for approximately 50–80% of the pulmonary phenotypic variability in CF patients with the same *CFTR* genotype (homozygous F508del) (Vanscoy et al., 2007).

## Genetic Approaches

**Figure 1:**
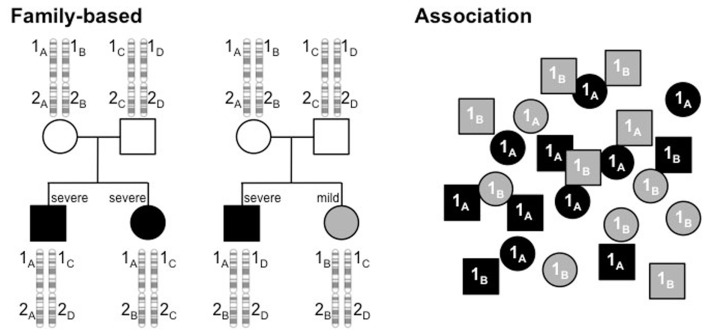
**Linkage analysis tracks alleles of polymorphisms through families to determine if an allele is linked to a phenotype**. In this example, alleles of gene 1, 1_A_, 1_B_, 1_C_, and 1_D_, track with severity (black, severe; gray, mild), showing concordant genotypes between siblings with similar phenotypes (left pedigree) and discordant genotypes when phenotypes are dissimilar (right pedigree). In contrast, genotype and phenotype show no relationship at polymorphism 2. Association studies examine a population of unrelated individuals to determine if particular alleles of a polymorphism are found in different proportions, depending on the disease profile. In the example here, alleles 1_A_ and 1_B_ have equal frequencies in the population, but 1_A_ is much higher in the severely affected subjects (black) and 1_B_ higher in the mildly affected subset (gray).

With a genetic component established, the next task at hand was to identify the genes responsible. There are two fundamental strategies by which to accomplish this. One requires family information and is often referred to as linkage analysis. Through this approach, one determines whether a polymorphism’s genotype is concordant in siblings with similar clinical profiles, discordant when clinical features are discordant or show no pattern. The other approach is association, determining if particular alleles of a polymorphism are distributed randomly among patients or have skewed distributions that track with clinical characteristics. These two approaches are outlined in Figure 1 and the findings that these strategies have produced are listed in Table 1 with several examples described in more detail below.

**Table 1:** **Summary of published cystic fibrosis pulmonary modifiers**.

Gene/locus	Genes involved	Variant aliases	Variant position (rs no.)	Phenotypes tested	Association *p*-value	Source *n* (reference)	Replication *n* (reference)	Tested, not replicated *n* (reference)
8.1AH	*LTA* *TNF*	+252 A > G −308 G > A	909253 1800629	FEV_1_ % pred Chronic *P. aeruginosa* colonization	<0.04 0.99	404 (Corvol et al., 2012)		
	*HSP70-2* *RAGE*	1267 A > G −429 T > C	106158 1800625					
8.1MHC	*AGER* *HSP70-2*	−429 T > C 1267 A > G	106158	Age at onset of colonization Frequency of colonization	0.036 0.012	72 (Laki et al., 2006)		
	*TNFA*	G-308A						
11p13	*APIP* *EHF*		12793173	FEV_1_ % pred (adjusted)	3.34 × 10^−8^	1,978 (Wright et al., 2011)	557 (Wright et al., 2011)	
19q13	*hCFM1*	APOC2, D19S219, D19S112 haplotype		FEV_1_ % pred	0.779	197 sib pairs (Zielenski et al., 1999)		
*A1AT*	*SERPINA1*	1237 G > A	11568814	FEV_1_ % pred CXR score Age at onset of *P. aeruginosa*	0.368 0.813 0.146	157 (Mahadeva et al., 1998b)	716 (Frangolias et al., 2003)	124 (Henry et al., 2001) 320(Courtney et al., 2006) 808 (Drumm et al., 2005)
		S allele Z allele	17580 28929474	FEV_1_% pred CXR score Age at onset of *P. aeruginosa*	0.043 0.127 0.899	157 (Mahadeva et al., 1998b)	215 (Doring et al., 1994) 79 (Mahadeva et al., 1998a)	124 (Henry et al., 2001) 269 (Meyer et al., 2002) 808 (Drumm et al., 2005)
*ABCC1*	*MRP-1*	4741 C > G	504348	Age at onset of *P. aeruginosa* Age at which FEV_1_ < 60%	0.0644 <0.05	203 (Mafficini et al., 2011)		
				FEV_1_ % pred	0.52			
*ABO*		T99T 21404 C > A	8176719 8176720 1053878	Pulmonary disease severity Age at onset of *P. aeruginosa*	No association No association	778 (Taylor-Cousar et al., 2009)		
		R176G	7853989					
		21583 T > A	8176740					
		H219H	8176741					
		P227P	8176742					
			816750					
		66119 G > A	8176472					
*ACE*		Insertion or deletion		Age of first *P. aeruginosa* infection	0.9	261 (Arkwright et al., 2003)		808 (Drumm et al., 2005)
				Age at which FEV_1_ < 50%	0.03 (0.04)^§^			
				Age of death	No association			
*ADRB2*		Arg16Gly	1042713	FEV_1_ % pred FVC	<0.05 <0.05	126 (Buscher et al., 2002)		808 (Drumm et al., 2005)
				Flows at lower lung volumes	<0.01			
				5 year decline in pulmonary function	<0.01			
		Gln27Glu Thr164Ile	1042714 1800888	Bronchodilator responses to albuterol	NS			
				Pulmonary function	Reduced			
*AGER*		−429T > C	1800625	FEV1 Kulich CF-specific percentile z-score	0.02 0.03	967 (Beucher et al., 2012)		
				KNoRMA	0.03			
*AGTR2*			1403543	FEV_1_ % pred (adjusted)	1.61 × 10^−5^	1,978 (Wright et al., 2011)		557 (Wright et al., 2011)
*AHRR*			12188164	FEV_1_% pred (adjusted)	5.92 × 10^−4^	1,978 (Wright et al., 2011)		557 (Wright et al., 2011)
*C3*		31778 G > A	393770	FEV_1_ % pred	0.75 (0.05)[Table-fn tfn2]	755 (Park et al., 2011)		
		4023 T > G	11569393		0.66 (0.03)[Table-fn tfn2]			
		39718 G > A	7257062		0.78 (0.52)[Table-fn tfn2]			
*CD14*		−159 C > T		Pulmonary disease severity	No association	105 (Faria et al., 2009)		
*CDH8*			11645366	FEV_1_ % pred (adjusted)	1.23 × 10^−5^	1,978 (Wright et al., 2011)		557 (Wright et al., 2011)
*CEACAM3*	*19q13*		6508999–10414823	Disease severity	0.0469	37 nuclear families (Stanke et al., 2010)		
*CEACAM6*	*19q13*		1549960–11548735	Disease severity	0.0106	37 nuclear families (Stanke et al., 2010)		
*CFB*		7680 A > G	537160	FEV_1_ % pred	0.50 (0.83)[Table-fn tfn2]	755 (Park et al., 2011)		
		10858 A > G	2072633		0.68 (0.74)[Table-fn tfn2]			
*CLCN2*	*CLC-2*	−693 A > G		FEV_1_ % pred	0.72	74 (Blaisdell et al., 2004)		
		358 G > C			0.32			
		427 A > G			0.32			
		1089 T > C			0.21			
		1909 G > C			0.22			
*DCTN4*		Any missense variant	11954652	Age at onset of chronic *P. aeruginosa* infection	0.05 0.002	91 (Emond et al., 2012)		
			35772018	Age of first *P. aeruginosa* infection	0.01		645 (Emond et al., 2012)^⊙^	
				Age at onset of chronic *P. aeruginosa* infection	0.004		530^○^	
				Age at onset of mucoid *P. aeruginosa* infection	0.03			
				Time from first detection of *P. aeruginosa* infection to mucoid *P. aeruginosa*	0.01			
*DEFB1*		Frequent polymorphisms		Age of first *P. aeruginosa* infection	No association	210 (Vankeerberghen et al., 2005)	62 (Segat et al., 2010)	224 (Tesse et al., 2008) 92 (Crovella et al., 2011)^+^
				FEV_1_%	No association			
*DEFB4*		Genomic copy number (2–12) of repeat unit		Pulmonary disease (mean and current FEV_1_, mean and current FVC)	No association	355 (Hollox et al., 2005)		
*EDNRA*		6672 G > C	5335	Pulmonary function (FEV_1_)	0.002	1,577 (Darrah et al., 2010)		
*EEA1*			4760506	FEV_1_ % pred (adjusted)	6.77 × 10^−6^	1,978 (Wright et al., 2011)		557 (Wright et al., 2011)
*FCGR2*	*Fc*γ*RII*	R131H		Chronic *P. aeruginosa* colonization	0.042	167 (De Rose et al., 2005)		
*FUT2*		G428A	601338	Impairment of lung function (FEV_1_)	0.569	806 (Taylor-Cousar et al., 2009)		
*FUT3*		T59G	28362459	Impairment of lung function (FEV_1_)	0.544	707 (Taylor-Cousar et al., 2009)		
		T202C	812936		0.491			
		C314T	778986		0.615			
		T1067A	3894326		0.792			
*GCLC*		(GAG)_n_		FEV_1_ % pred	0.097	440 (McKone et al., 2006)		
					0.001 (mild)			
					0.533 (severe)			
*GSTM1*		GSTM1*0/ GSTM1*0		FEV_1_ % pred	0.16	53 (Hull and Thomson, 1998)	194 (Baranov et al., 1996)	146 (Flamant et al., 2004)
				Chrispin–Norman score	0.02			808 (Drumm et al., 2005)
				Shwachman score	0.04		60 (Korytina et al., 2004)	
				Positive for *P. aeruginosa*	0.12			
				No. of ΔF508 homozygotes	0.43			
*GSTM3*		GSTM3*A	1799735	FEV_1_	0.01	146 (Flamant et al., 2004)		
		GSTM3*B		FVC	0.002			
*GSTP1*		1375 A > G	947894	Spirometry	NS	146 (Flamant et al., 2004)	808 (Drumm et al., 2005)	60 (Korytina et al., 2004)
		I105V						
*GSTT1*		GSTT1*0/		Spirometry	NS	146 (Flamant et al., 2004)		
		GSTT1*0						
*HFE*		C282Y and/or H63D	1800562 and/or 1799945	Positive for *P. aeruginosa*	0.81	82 (Pratap et al., 2010)		
				FEV_1_% pred	0.03			
				FVC% pred	0.02			
				Annual change in FEV_1_% pred	0.003			
				Annual change in FVC% pred	0.001			
*HLA*		DRA	9268905	FEV_1_ % pred (adjusted)	1.42 × 10^−5^	1,978 (Wright et al., 2011)	557 (Wright et al., 2011)	
		DR4		Chronic *P. aeruginosa* colonization	≤0.03	98 (Aron et al., 1999)		72 (Laki et al., 2006)
		DR7/DQA*0201		Chronic *P. aeruginosa* colonization	<0.03			
*HMOX1*		11354 A > G	2071749	FEV_1_ % pred	0.01 (0.29)[Table-fn tfn2]	755 (Park et al., 2011)		
		4613 A > T	2071746		0.40 (0.03)[Table-fn tfn2]			
*IFNG*	*IFN*γ	+874 A > T		Age of first *P. aeruginosa* infection	No association	261 (Arkwright et al., 2003)		
				Age at which FEV_1_ < 50%	0.09			
				Age of death	No association			
*IFRD1*		57460 C > T	7817	Cross-sectional measures of lung function	0.004 (0.0168)^£^	320 (Gu et al., 2009)		
				Longitudinal measures of lung function	0.016 (0.0187)^£^			
				FEV_1_% pred (adjusted)	No association	1,978 (Wright et al., 2011)		
		47556 G > T	3807213	Longitudinal measures of lung function	0.080			
		38923 C > T	6968084	Cross-sectional measures of lung function	0.082			
*IL8*			2227306	Pulmonary disease severity	0.19	737	385 (Hillian et al., 2008)	
			2227307		0.04	727		
			2227543		0.06	732	329 (Corvol et al., 2008)^♦^	
		−251 A > T	4073		0.07	733 (Hillian et al., 2008)		
*IL-10*		−592 CC/-	1800872	Pulmonary function decline	No association	261 (Arkwright et al., 2003)		
		−592 CC/TA		Age of first *P. aeruginosa* or *B. cepacia* infection	No association			
				Age of death	No association			
		−1082 G > A	1800896	Colonization with *A. fumigatus*	0.06 (0.03)^§^ 0.02 (0.01)^§^	378 (Brouard et al., 2005)		808 (Drumm et al., 2005)
				Development of ABPA	No association			
				Colonization with *P. aeruginosa*				
*KRT8/KRT18*			1907671	Disease severity	Associates	49 (24 sib pairs) (Stanke et al., 2011)		
			4300473		Associates			
			8608		Associates			
		7952 T > C	2035875		0.00131			
			1907671-4300473- 2035878-2035875 haplotype	Disease severity	0.0051			
			2638526	Disease severity	NS			
			2070876		NS			
*KRT19*		c.90T > C	11550883	Effective specific airway resistance	0.0093	95 (Gisler et al., 2012)		
		c.179G > C	4602		0.0052			
			11550883-4602 haplotype		0.0097			
*MASP-2*		Exon 3 A > G, D120G	72550870	Pulmonary function	No association	112 (Carlsson et al., 2005)	109 (Olesen et al., 2006)	
				Need for transplantation	No association			
				Colonization with *P. aeruginosa*	0.04			
				Lung function in patients colonized with *S. aureus*	0.04			
*MBL2*		X1 – B (A > G) X1 – C (A > G) X1 – D (C > T) (*A/A, A/O, O/O*) −221 G > C (*X/Y*)	1800450 1800451 5030737 7096206	FEV_1_% FVC% Age of onset of *P. aeruginosa*	0.003 0.03 0.07	149 (Garred et al., 1999)	164 (Gabolde et al., 1999) 179 (Yarden et al., 2004) 298 (Davies et al., 2004)^Θ^ 47 (Trevisiol et al., 2005)[Table-fn tfn9] 135 (Choi et al., 2006) 254 (Buranawuti et al., 2007)	112 (Carlsson et al., 2005) 260 (Davies et al., 2004)^Θ^ 47 (Trevisiol et al., 2005)[Table-fn tfn9] 808 (Drumm et al., 2005) 105 (Faria et al., 2009) 788 (McDougal et al., 2010) 123 (Olesen et al., 2006)
				Lung function	No association	112 (Carlsson et al., 2005)	105 (Faria et al., 2009)	
		−550 G > C (*H/L*)		Colonization	No association			
*MIF*		−794 presence of absence of 5-CATT		Colonization with *P. aeruginosa*	0.004	167 (Plant et al., 2005)		
				Colonization with *S. aureus*	0.50			
				Colonization with *Candida*	0.36			
				FEV_1_ ≥ 80%	0.14			
*NOS1*		(AAT)_9–15_		Colonization with *P. aeruginosa*	0.0358	75 (Grasemann et al., 2000)	40 (Grasemann et al., 2002)	
				Mean FE_NO_	0.027			
		(GT)_18–36_		Colonization with *A. fumigatus*	0.8505 0.025	59 (Texereau et al., 2004)		
				5 year decline of pulmonary function				
*NOS3*		894 G > T		FE_NO_	0.07 (0.02 in females)	70 (Grasemann et al., 2003)		
				FEV_1_	0.08 (in females)			
				Colonization with *P. aeruginosa*	<0.05			
		T5220G	1799983	Impairment of lung function (FEV_1_)	0.54	808 (Drumm et al., 2005)		
*PPP2R1A*		c.*465T > A	2162779	Functional residual capacity	0.0033	95 (Gisler et al., 2012)		
*PPP2R4*		c.−185A > C	3118625	FEV1	0.0048	95 (Gisler et al., 2012)		
				Lung clearance index	0.0059			
				Effective specific airway resistance	0.0064			
*SCNN1B*	*ENaC*β	T313M 938 C > T G589S 1765 G > A		Disease severity		56 (Viel et al., 2008)		
*SCNN1G*	*ENaC*γ	L481G 1442 T > A V546I 1636 G > A	5735–5723 haplotype	Disease severity		56 (Viel et al., 2008)		
*SERPINA3*	*ACT, A1ACT*	T-15A	4934	FEV_1_% pred	0.04	157 (Mahadeva et al., 2001)		
				Radiography score	0.03			
*SFTPA1*		6A^3^ (and 6A^3^/1A^1^ haplotype)		FEV_1_% pred	0.01	135 (Choi et al., 2006)		
				DLCO	0.10			
				ATS score	0.006			
				AMA score	0.02			
				Dyspnea score	0.20			
				Physical score	0.002			
				Severity score	0.005			
*SFTPA2*		1A^1^ (and 6A^3^/1A^1^ haplotype)		FEV_1_% pred	0.009	135 (Choi et al., 2006)		
				DLCO	0.13			
				ATS score	0.007			
				AMA score	0.06			
				Dyspnea score	0.07			
				Physical score	0.12			
				Severity score	0.10			
*SLC8A3*			12883884	FEV_1_% pred (adjusted)	1.20 × 10^−6^	1,978 (Wright et al., 2011)		557 (Wright et al., 2011)
*SLC9A3*		521096 C > T	4957061	Age of first *P. aeruginosa* infection	0.02	1,004		
				Decline of lung function (FEV_1_)	0.05	752 (Dorfman et al., 2011)		
*SNAP23*		c.267-9T > C	9302112	FEF_50_	0.0088	95 (Gisler et al., 2012)		
				Functional residual capacity	0.011			
				Volume of trapped gas	0.0043			
*TGFB1*		codon 10 C29T	1800470	Age at which FEV_1_ < 50% Age at which FVC < 70%	<0.02 <0.005	171 (Arkwright et al., 2000)*	261 (Arkwright et al., 2003)	118 (Brazova et al., 2006) 1,978 (Wright et al., 2011)
		codon 25 G74C	1800471	Age at which FEV_1_ < 50% Age at which FVC < 70%	NS NS		808 (Drumm et al., 2005)*	
		C-509T	1800469	Impairment of lung function (FEV_1_)	0.006	808 (Drumm et al., 2005)	498 (Drumm et al., 2005) 329 (Corvol et al., 2008) 105 (Faria et al., 2009) 472 (Bremer et al., 2008)	254 (Buranawuti et al., 2007)
*TLR4*		D299G	4986790	Mean FEV_1_% pred	0.55	100 (Urquhart et al., 2006)		
				Mean FVC% pred	0.52			
				Age of first *P. aeruginosa* infection	0.78			
				Chrispin–Norman X-ray score	0.16			
		2688 G > A	10759931	Rate of change of FEV_1_% pred per year	0.12			
				FEV_1_ % pred	0.84 (0.55)[Table-fn tfn2]	755 (Park et al., 2011)		
*TLR5*		R392X	5744168	Mean FEV_1_ % pred	0.77	2219 (Blohmke et al., 2010)		
*TNFA*	*TNFa*	G-308A (TNF2)	1800629	Mean FEV_1_% pred Mean Chrispin–Norman X-ray score	0.02 0.17	53 (Hull and Thomson, 1998) 180 (Yarden et al., 2005)		261 (Arkwright et al., 2003) 180 (Yarden et al., 2005) 53 (Schmitt-Grohe et al., 2006)
				Mean Shwachman score	0.17			
				No. positive for *P. aeruginosa*	0.72			808 (Drumm et al., 2005)
		C-851T		Mean FEV_1_% pred Age of first *P. aeruginosa* infection	0.25 0.60			
		G-238A		Mean FEV_1_% pred Age of first *P. aeruginosa* infection	0.8 0.64			
		+691g ins/del		Mean FEV_1_% pred Age of first *P. aeruginosa* infection	0.008 0.018			
*TNFR1*	*TNFRSF1A*	intron 1 haplotype		Disease severity	Associates	37 sib pairs (Stanke et al., 2006)		

*^§^The number in parenthesis indicates the *p*-value for the association found in F508del homozygotes*.

*^
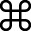
^Only multivariate *p*-values are reported. The number outside the parenthesis is the *p*-value for pediatrics and the number in parenthesis is the *p*-value for adults*.

*^⊙^The association of missense variants with age at first *P. aeruginosa*-positive culture and age at onset of chronic *P. aeruginosa* was replicated in a population of only European American patients*.

*^○^The association of missense variants with age at first *P. aeruginosa*-positive culture and age at onset of chronic *P. aeruginosa* was replicated in a population excluding patients with non-European ancestry*.

*^+^Found that only the c.−20G > A SNP associated with disease severity*.

*^£^The number in parenthesis indicates the *p*-value after a Bonferroni correction*.

*^♦^Found that −251 TT, +396TT, and +781CC may be associated with an earlier occurrence of chronic *P. aeruginosa* colonization, which is an indicator of disease severity, but this was not examined in the study by Hillian et al. (2008)*.

*^Θ^The association of MBL2 deficiency alleles with indicators of pulmonary disease severity was replicated in a population of 298 adults, but refuted in a population of 260 children*.

*^
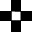
^The Trevisiol et al. (2005) study replicated an association of MBL2 deficiency alleles with pulmonary function, but not with PA colonization*.

*The study by Arkwright et al. (2000) found the severe variant at codon 10 to be T/T, but the study by Drumm et al. (2005) found the severe variant to be C/C at codon 10. A more detailed discussion of the *TGB1* association with CF can be found in the text.

**Figure 2:**
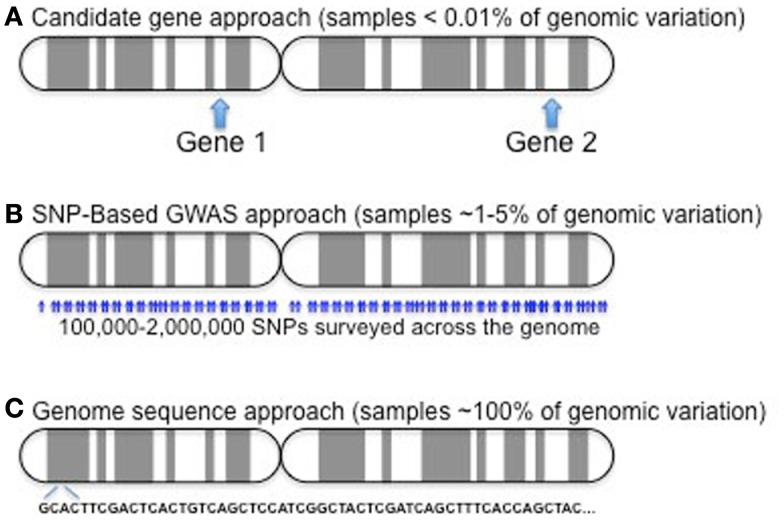
**Candidate gene approaches (A) have only involved a few variants in one to several dozen genes**. Given a genome of roughly 25,000 genes, this represents a very small sampling (∼0.01% or less). GWAS **(B)** samples a much larger component of the genome, probing more than 90% of the genes, but it still only examines less than 5% of the over 50 million reference SNPs (http://www.ncbi.nlm.nih.gov/mailman/pipermail/dbsnp-announce/2012q2/000123.html) curated as of June, 2012. As costs come down, exome (not shown) and whole-genome sequencing **(C)** provide the potential to capture all variation in study subjects.

The vast majority of studies have been of the association design, predominantly due to the small number of families with multiple, affected children. These studies have evolved over time; cost and time restricted most early studies to screen for potential disease-modifying genes by candidate gene approaches with later studies utilizing array-based methods and soon whole-genome sequencing will be the state of the art. These three approaches are compared in Figure 2.

### Phenotypic considerations

As lung disease is the major source of CF-related mortality, most studies have focused on some measure of lung function as a phenotype to examine for association. As most CF care centers carry out standard pulmonary function tests, spirometry has most commonly been used. Other tests may, in fact, be more specific for particular modifying functions, such as lung clearance index, but these are not as widely used and thus less practical for multi-center studies.

### Candidate genes

Candidate genes are those suspected to have a role in some aspect of CF pathophysiology and variants in those genes are then tested for association with disease manifestations. Those traits may be represented by a continuum of values (lung disease severity, for example) or discrete traits, such as the occurrence of intestinal obstruction. Candidate gene selections for study involved many areas because of the complex pathophysiology of CF, including bacterial infections, inflammation, and lung remodeling/deterioration. This approach yielded multiple reports of putative modifiers of the CF pulmonary phenotype. For example, mannose-binding lectin (*MBL*), a gene involved in innate immunity, was one of the first potential modifier genes described. Low-expressing *MBL* alleles were found to associate with a more severe pulmonary disease course than those with higher expression (Garred et al., 1999). *HLA* haplotypes were also investigated as modifiers due to the role of the genes in this complex in innate defense and inflammation. Carriers of the *HLA* II DR7 haplotype were found to have a higher incidence of *P. aeruginosa* colonization (Aron et al., 1999).

Polymorphisms within cytokines and other inflammatory mediators were investigated as potential modifiers of CF pulmonary disease due to their role in immune response as well. Tumor necrosis factor alpha (TNFα) is a pro-inflammatory cytokine that is stimulated by NF-κB as a first line of defense against infection. The minor allele of a *TNF*α promoter polymorphism associated with worse pulmonary function in a small set of CF patients (Hull and Thomson, 1998). Interestingly, the *TNF*α minor allele that associated with a worse CF prognosis was also associated with an increase in mRNA expression level when measured using a reporter construct (Wilson et al., 1992). Interleukin-10 (IL-10), an anti-inflammatory cytokine was also investigated. Like *TNF*α, an *IL-10* promoter polymorphism was also associated with differences in IL-10 expression (Turner et al., 1997). In this case, the lower expressing *IL-10* allele was associated with worse CF disease. These studies supported a model in which higher levels of the pro-inflammatory cytokine TNFα, and lower levels of the anti-inflammatory cytokine IL-10 contribute to more severe CF lung disease.

### Challenges of early candidate gene modifier studies

Early studies that attempted to identify potential modifiers were challenged by small numbers of study subjects. Typically, pulmonary function data using standard spirometry are not available on children younger than age 6, and multiple measures over time are needed to assess a subject’s trajectory, as an indicator of current and future disease severity. Nonetheless, numerous studies compared pulmonary function of subjects over a range of ages, statistically adjusting for age. Younger patients were included in order to maximize participation, but epidemiologic studies indicated that much of the pulmonary phenotypic variability was not present until after puberty (Zemel et al., 2000).

An additional constraint is that not all mutations in *CFTR* have the same consequences on protein function and thus it is likely to confound interpretation if *CFTR* genotype is not accounted for. Consequently, after limiting to patients with sufficient lung function measurements and comparable *CFTR* genotypes, the number of available subjects is low, making it unfeasible for any single center to carry out an association study that would have the statistical power to detect anything but a very major effect of a modifier gene.

### Consortium approaches

The ability to effectively carry out genetic studies is limited by numbers of subjects. As a means to increase numbers, the European CF Twin and Sibling Study mentioned earlier was conceived and compared morphometric and pulmonary function indices of sib pairs. Using lung function measurements from patients in North America and Europe, this study was the first to compare lung function using a CF population for reference (Mekus et al., 2000).

Subsequently, the CF Gene Modifier Study (GMS) was conceived in 1999 to carry out a genetic study on a large group of patients for which longitudinal lung function data were available and genotype was restricted. In its inception, the study design was to use a candidate gene approach to search for potential genetic modifiers of CF pulmonary disease. The unique study design reduced genetic heterogeneity by using only patients who were homozygous for F508del (commonly referred to as ΔF508), and maximized the number of patients available by including patients from CF centers nationwide, comparing the most mild and most severe patients for differences in allele or genotype frequencies of single nucleotide polymorphisms (SNPs) or other gene-associated variants as markers of potential modifier genes.

Phenotypic categories of disease severity were defined using a patient’s forced expiratory volume in 1 s (FEV_1_), a pulmonary function index based on age, sex, and height, and used clinically to monitor CF disease progression and therapeutic efficacy. Subjects with FEV_1_ values in the upper quintile were classified as “mild” and those in the lower quintile as “severe.” Those subjects surviving beyond the age of 34 were classified as mild regardless of pulmonary function, as they represented the upper quintile of their birth cohort (Schluchter, 1992; Schluchter et al., 2002). DNA was obtained from these individuals and genotyped for a variety of variants in or near genes that were considered candidate modifiers.

In the initial candidate gene approach, 1,064 SNPs were tested in over 300 genes/gene regions that were chosen in the following ways: (1) they were SNPs that had previously been reported in the literature as associating with CF phenotype, (2) they were SNPs that were reportedly associated with similar pulmonary disease phenotypes, (3) they were genes that were known to play a key role in CF pathophysiology (Drumm et al., 2005).

Experience using this approach has shed light on the challenges involved in conducting modifier studies. Early studies struggled to achieve statistical power due to small sample sizes. Long and Langley (1999) calculated that the sample size must include at least 500 individuals in order to detect a causative polymorphism and for its association to be replicable. To accommodate the ability to replicate and maximize power, the GMS expanded to a North American Consortium that included a family-based genetic study at the Johns Hopkins University and a population-based study of Canadian CF patients being led by investigators at the University of Toronto and the Hospital for Sick Kids (Taylor et al., 2006). This consortium grew from the need to increase sample size and carry out replication studies and demonstrated its utility in a report that showed variants in the *TGFB1* gene associate with pulmonary disease (Drumm et al., 2005) (discussed in more detail below).

The union of the three large studies provided a cohort of unprecedented size for studying modifiers of a single gene disorder, but also presented logistical issues due to the nature of the designs as each group had developed their own methods for assessing pulmonary phenotypes. Kulich et al. (2005) generated CF-specific reference equations for FEV_1_ that compare a CF subject’s lung function to CF subjects of the same age, sex, and height, as a more appropriate reference than the non-CF population and those values, adjusted for survival, were used to develop a phenotypic index that all three designs could incorporate.

The candidate gene approach showed the effectiveness of genetic studies, but a limitation is that it does not identify genetic locations other than those suspected to influence disease. That is, it will not detect modifying genes or pathways beyond those involved in our limited understanding of the disease. Understanding the functional effects of a modifier and its protein product fuel future studies to provide mechanistic insight of disease pathophysiology and how it might be dealt with (Cutting, 2010).

## Associating Genes and Insight into their Modifying Mechanisms

One of the powerful attributes of genetics is that it allows one to identify clinically relevant genes, proteins, or pathways by virtue of the effect that variation in the gene produces on a clinical trait. However, the mechanisms by which genetic variation acts on the phenotype is not necessarily obvious. Thus, for any associating gene an obligatory step is to carry out functional studies to understand how it imparts its effect on disease presentation or outcome. Some examples are given below.

### Associating genes: MBL

Mannose-binding lectin is a serum protein involved in innate immunity. MBL enhances phagocytosis of infectious organisms, especially during infancy, when adaptive immune response is immature (Eisen and Minchinton, 2003). Variant alleles that decrease MBL serum levels increase risk for many different infections (Garred et al., 1995, 1997; Summerfield et al., 1995, 1997) and have been shown to play a role in autoimmune diseases (Davies et al., 1995; Graudal et al., 1998). MBL has been suggested to regulate inflammatory responses, perhaps by delaying one of the first steps in inflammation or by reducing the levels of inflammatory cytokines (Jack et al., 2001). *MBL* is an attractive CF modifier candidate because it protects against infection and has some role in modulating inflammation.

Three amino acid substitutions in exon 1 (alleles B, C, and D) each contribute to decreased MBL plasma concentrations and are collectively referred to as 0, or null, alleles with the functional allele, containing none of the above variants, designated *A*. There are also variants with quantitative effects on mRNA expression, termed *X*, that also result in low MBL serum levels. Genotypes resulting in low MBL levels are designated low-producing or deficient alleles, but there are also genotype combinations associated with high and intermediate serum levels of MBL as well. Using the rationale that MBL protects against bacterial infection or somehow suppresses inflammation, then *MBL* deficiency alleles would be predicted to associate with a more severe CF lung disease.

In support of such a model, Garred et al. (1999) found that patients with higher expression *MBL* genotypes had a higher FEV_1_ and forced vital capacity (FVC). In other words, there was an additive effect of poor pulmonary function in the presence of an *X* allele. After further analysis, the cumulative adverse effects of low expression alleles were restricted to patients with chronic *P. aeruginosa* and were more pronounced in adults. MBL deficiency did not significantly associate with chronic colonization of *P. aeruginosa*. A study by Gabolde et al. found that cirrhosis of the liver was more common in CF patients carrying deficiency alleles, but other sources are conflicting about the association with CF liver disease (Gabolde et al., 2001; Bartlett et al., 2009; Tomaiuolo et al., 2009).

Several studies agree that *MBL* low expression alleles associate with lung function (Gabolde et al., 1999; Davies et al., 2004; Yarden et al., 2004; Trevisiol et al., 2005; Choi et al., 2006; Buranawuti et al., 2007; Dorfman et al., 2008), but there is no consensus as to whether this effect is only seen in patients colonized with *P. aeruginosa*, and whether a heterozygous genotype is sufficient to cause such impairment. Two studies found an association with chronic *P. aeruginosa* colonization (Trevisiol et al., 2005; McDougal et al., 2010), whereas others failed to detect an association between *MBL* alleles and colonization of any kind. Buranawuti et al. (2007) found that *MBL* high expression alleles predicted survival; the null genotype was underrepresented in adult populations and over represented in patients who died late in adolescence. This is consistent with multiple observations that the adverse effect of deficiency alleles is more pronounced in adults (Garred et al., 1999; Yarden et al., 2004; Buranawuti et al., 2007). In fact, a study by Davies et al. (2004) found no association between pulmonary function and *MBL* genotype in children. Despite replications, not all studies have detected associations between *MBL* alleles and lung disease severity (Carlsson et al., 2005; Drumm et al., 2005; Faria et al., 2009; McDougal et al., 2010).

### Associating genes: *TGFB1*

As alluded to above, the first significant association identified by the consortium approach demonstrated that severity of pulmonary disease tracked with variants in the *TGFB1* gene (Drumm et al., 2005). *TGFB1* encodes transforming growth factor beta-1 (TGFβ1), a protein with complex function, involved in several cellular processes from differentiation and proliferation to innate immunity, and has been studied in relation to many disorders including Alzheimer’s disease, cancer, Marfan disease, and heart disease (Waltenberger et al., 1993; Yamamoto et al., 1993; Dickson et al., 2005; Brooke et al., 2008). Interest in investigating *TGF*β*1* as a potential modifier of CF pulmonary disease stemmed from both its biologic plausibility, and its identification as a modifier of asthma and chronic obstructive pulmonary disease (COPD) (Pulleyn et al., 2001; Celedon et al., 2004; Silverman et al., 2004; Wu et al., 2004).

TGFβ1 is biologically relevant to CF for several reasons. Leukocytes secrete TGFβ1 in response to infectious agents. TGFβ1 participates in the immune process by regulating the production of cytokines, and is generally thought to be pro-inflammatory in nature (Omer et al., 2003). TGFβ1 also increases the formation of extracellular tissue during injury repair by increasing production of connective tissue by altered gene regulation (Bartram and Speer, 2004). Post-injury repair in the lung is a delicate balance; inadequate remodeling leads to poor wound healing, whereas excessive remodeling leads to pathogenic fibrosis and scarring. There is strong evidence to suggest that the difference between these outcomes is at least in part related to *TGF*β*1* expression levels (Bartram and Speer, 2004).

Variation in *TGF*β*1* has been shown to modify asthma and COPD. A variant in the promoter region (C-509T), thought to be associated with increased *TGF*β*1* expression, was studied as a potential contributor to asthma disease severity. In two separate studies homozygosity for the T allele (associated with increased TGFβ1 production) was found to be more common among severe asthmatics when compared to mild asthmatics or healthy controls (Pulleyn et al., 2001; Silverman et al., 2004). Variation in codon 10 was studied in patients with COPD. In this case, the allele associated with increased TGFβ1 production was found more commonly in control patients, suggesting a protective role for TGFβ1 in COPD (Wu et al., 2004). Contrasting with associations found in asthma patients, the T allele of -509 was more prevalent in those with mild COPD (Celedon et al., 2004).

The *TGF*β*1* variants that have been implicated in other airway diseases have become a source of interest in CF as well. A study by Arkwright et al. (2000) found that the T allele (high producer genotype) in codon 10 associated with more rapid deterioration in lung function, while the genotype at codon 25 did not correlate with survival or lung function. Another study confirmed the codon 10 association found by Arkwright but interestingly, it was the C allele (low producer genotype) that prevailed in severe patients (Drumm et al., 2005). This finding, replicated in a second population of 498 patients, is counterintuitive given the protective role of TGFβ1 in COPD. The same study, by Drumm et al. found that the -509 T allele also associated with a severe pulmonary phenotype, which is the same adverse effect seen in asthma populations. There have been several attempts to resolve these conflicting data (Arkwright et al., 2000, 2003; Drumm et al., 2005; Brazova et al., 2006; Buranawuti et al., 2007; Bremer et al., 2008; Corvol et al., 2008; Faria et al., 2009), but only one study has used a relatively large cohort to accommodate the statistical power needed. It found that a haplotype of a 3′ C allele (rs8179181), -509 C, and codon 10 T associated with improved lung function to a greater degree than any SNP alone (Bremer et al., 2008). It would appear from these studies that CF more closely mimics the type of disease seen in asthma and that the same polymorphisms may be protective or adverse, depending on the genetic and environmental context.

### Associating genes: *IFRD1*

Gu et al. (2009) applied a novel strategy by pooling equal amounts of DNA from similarly affected subjects into “mild” and “severe” pools and examined 320 patients in the GMS population (160 with severe lung disease, 160 with mild lung disease) with much lower cost and time than the other efforts. By quantifying the signal for each allele (rather than a yes/no output) the genotyping arrays were used to estimate allele frequencies in the pools. Discordant allele frequencies were identified between the pools using this strategy (Gu et al., 2009) and indicated that alleles of *IFRD1* may contribute to pulmonary disease severity. In a subsequent study, however, *IFRD1* variants did not significantly associate with lung disease (Wright et al., 2011).

The IFRD1 protein acts in a histone deacetylase (HDAC)-dependent manner to regulate gene expression (Vietor et al., 2002) and the *IFRD1* gene is up-regulated during cell differentiation and regeneration in response to stress (Vietor and Huber, 2007). Previous studies found high expression in human blood cells (SymAtlas, 2008) and Gu et al. found highest expression in neutrophils, where up-regulation occurs during the final differentiation steps (Ehrnhoefer, 2009; Gu et al., 2009). The authors suggested that IFRD1 modulates CF lung disease through the regulation of neutrophil effector function, but that other explanations, involving different cell types, should not be ignored.

## Genome-Wide Association Studies

Although the cost of large-scale genotyping had fallen more than a 1000-fold since these studies were initiated, genome sequencing was still well out of range by price and feasibility. Thus, it became feasible to think about whole genome, or genome-wide association studies (GWAS). A GWAS would rapidly interrogate hundreds of thousands of SNPs for association in large populations (Manolio, 2010) without bias imposed by pre-existing models and provide the opportunity to identify novel genes, regulatory loci, and pathways not previously considered. The disadvantage to testing so many variants is that there are statistical penalties that increase as the number of comparisons rises, and thus power is a major limitation (Cutting, 2010). This is less of a concern if the effect of a locus is large, but as common population variants are being examined in these studies, it is likely that the effects of any one locus are not large, perhaps with each accounting for only a few percent of the variation, for example (Long and Langley, 1999). It is an important concept to understand that these studies are conceptually analogous to those designed to find disease-causing genes, which would have major effects if they do, in fact, cause disease.

### GWAS-identified associations

In a combined GWAS and family-based (linkage) study, 3,467 CF patients were tested for associations between lung disease severity and more than half a million SNPs (Wright et al., 2011). To accommodate the various study designs and data acquisition protocols, yet another method to examine pulmonary function, with age-specific CF percentile values of FEV_1_ (Kulich et al., 2005; Taylor et al., 2011), was developed and which accounted for mortality and longitudinal changes. With this phenotype and over 500,000 common genetic variants to assess for association, two new loci, one on chromosome 11p13 and one on chromosome 20q13 were identified as having variants that associate with lung function in CF.

The region on chromosome 11p13 of most significant association lies between two annotated genes, *APIP* and *EHF*. *APIP* encodes Apaf-1-interacting protein and EHF is a member of the epithelial-specific Ets transcription factors, both of which provide interesting candidates as disease modifiers, but through very different models, all of which must yet be worked out. It is important to understand that despite the power of genetics to identify such disease-relevant locations in the genome, it does not provide information regarding mechanisms and these must be examined empirically. APIP, for example, has been shown to suppress apoptosis in the presence of hypoxia (Cho et al., 2007), a context experienced by CF tissues. At this point, it is not clear if the adverse allele provides less or greater activity than the protective allele, but one could construct models either way. For example, one hypothesis is that excessive anti-apoptotic activity, resulting from increased APIP, could prolong neutrophilic inflammation and therefore lead to more severe lung disease (Wright et al., 2011). Similarly, EHF is reported to serve as a regulator of epithelial cell differentiation under conditions of stress and inflammation (Tugores et al., 2001; Wright et al., 2011) and thus could be modeled to have very important effects during airway development or remodeling from disease-related damage. Finally, it must be considered that the modifying locus could be working at a distance, involving a regulatory site such as a transcriptional enhancer or non-coding RNA.

The other associating region on chromosome 20 was detected by linkage analysis and then refined by association. The linkage signal includes several genes including *MC3R*, encoding the melanocortin-3 receptor, *CBLN4* encoding cerebellin-like 4, *CASS4*, encoding Crk-associated substrate scaffolding (CASS) 4, and *AURKA*, encoding Aurora kinase A (Wright et al., 2011). With the exception of MC3R, which is a receptor involved in metabolic control, models to explain the other candidates are not presently clear.

Certainly functional studies will help sort out which genes in these associating intervals are responsible for their modifying effects, but these findings illustrate both the power and some of the challenges of genetic studies. On one hand, the unbiased approach provides the opportunity to identify novel disease modulators, but on the other hand identifying the source of the modifying effect and the mechanisms through with it acts are challenging tasks.

## The Impact of Disease-Modifying Genes

The implications of disease-modifying genes are multiple. First, understanding the genetic contribution to phenotypic variation has the potential to provide insight into prognosis. Second, understanding the mechanisms by which these genes and their alleles are exerting their effects will likely suggest new therapeutic approaches or ways to optimize existing ones. Third, it opens the door to personalized medicine, as a given patient’s treatment regimen could conceivably be developed around a genetic profile. Using inflammation as an example, one could imagine a patient whose modifier panel predicts a lessened inflammatory response, and another patient whose modifier panel predicts a heightened inflammatory response. Inflammation is part of the immune response that is necessary to fight infection, however its prolonged state in CF patients can cause lung damage. The patient with the heightened response may benefit from anti-inflammatory drugs earlier, and the patient with the reduced inflammatory response may benefit from increased antibiotic usage. Both are common treatments for CF, but they may be used more beneficially with the help of modifier identification and mechanistic understanding.

## Summary

Cystic fibrosis is a simple, Mendelian disorder with complex clinical manifestations that are consequences of *CFTR* genotype, environmental factors (Boyle, 2007), and heterogeneity throughout the entire genome. The discovery of genetic modifiers may help account for the broad spectrum of disease severity observed in patients, especially those with the same *CFTR* genotype. Modifying loci identified thus far each appear to contribute only a small percentage to overall disease profile and thus it is likely the combination of these variants in different permutations shape an individual’s outcome, an outcome that is also significantly influenced by non-genetic factors, as well as the interaction of genetic and non-genetic factors. There are few genes whose modifying effects withstand the test of replication and further studies must elucidate the role of each one in CF. Additional research about gene-environment interactions and gene–gene interactions will certainly demonstrate how complex these genetic effects are. With the careful use of candidate gene approaches and now, genome-wide scans (and soon whole-genome sequencing), it is realistic to believe that modifiers of CF disease will be identified and from which interventions tailored around an individual’s genetic profile will be developed. This fine-tuning of therapeutic strategies could contribute to better quality of life and ultimately, improved survival in CF.

## Conflict of Interest Statement

The authors declare that the research was conducted in the absence of any commercial or financial relationships that could be construed as a potential conflict of interest.

## Footnotes

^1^www.genet.sickkids.on.ca

^2^http://www.cftr2.org
